# Implementation of an improved dose‐per‐MU model for double‐scattered proton beams to address interbeamline modulation width variability

**DOI:** 10.1120/jacmp.v15i3.4748

**Published:** 2014-05-08

**Authors:** Liyong Lin, JiaJien Shen, Christopher G. Ainsley, Timothy D. Solberg, James E. McDonough

**Affiliations:** ^1^ Department of Radiation Oncology University of Pennsylvania Philadelphia PA; ^2^ Department of Radiation Oncology Mayo Clinic Phoenix AZ USA

**Keywords:** proton therapy, double scattering, output model

## Abstract

Because treatment planning systems (TPSs) generally do not provide monitor units (MUs) for double‐scattered proton plans, models to predict MUs as a function of the range and the nominal modulation width requested of the beam delivery system, such as the one developed by the MGH group, have been proposed. For a given nominal modulation width, however, the measured modulation width depends on the accuracy of the vendor's calibration process and may differ from this nominal value, and also from one beamline to the next. Although such a difference can be replicated in our TPS, the output dependence on range and modulation width for each beam option or suboption has to be modeled separately for each beamline in order to achieve maximal 3% inaccuracy. As a consequence, the MGH output model may not be directly transferable. This work, therefore, serves to extend the model to more general clinic situations. In this paper, a parameterized linear‐quadratic transformation is introduced to convert the nominal modulation width to the measured modulation width for each beam option or suboption on a per‐beamline basis. Fit parameters are derived for each beamline from measurements of 60 reference beams spanning the minimum and maximum ranges, and modulation widths from 2 cm to full range per option or suboption. Using the modeled modulation width, we extract the MGH parameters for the output dependence on range and modulation width. Our method has been tested with 1784 patient‐specific fields delivered across three different beamlines at our facility. For these fields, all measured outputs fall within 3%, and 64.4% fall within 1%, of our model. Using a parameterized linear‐quadratic modulation width, MU calculation models can be established on a per‐beamline basis for each double scattering beam option or suboption.

PACS number: 87.53.Qc

## I. INTRODUCTION

It is well known that proton therapy has the advantage of sparing normal tissue because of the finite proton range. The clinical utilization of proton therapy requires safe and efficient planning and delivery technologies. However, the calculation of output (dose/MU) is not supported by commercial proton therapy treatment planning systems (TPSs) due to the complexities of the beam delivery systems. Historically, output was, therefore, determined by measurement for each field prior to treatment. Although adequate, this undertaking unfortunately requires a significant amount of beam time and manpower.

Output calculation methods for different proton therapy systems, using either empirical models^1^, ^2^, ^3^, ^4^, ^5^, ^6^, ^7^ or Monte Carlo simulations,^8^, ^9^, ^10^ have been described in several publications. These methods have been used for MU determination and/or as independent checks of measured output. Although less sophisticated, the advantage of empirical models over Monte Carlo simulations is their explicit form.

An analytical expression for the depth‐dose of a spread‐out Bragg peak (SOBP) at infinite source‐to‐axis distance (SAD) was derived by Bortfeld and Schlegel in 1996.^(11)^ Kooy et al.^(1)^ extended Bortfeld and Schlegel's analysis to a specific model for the IBA (Ion Beam Applications, SA, Louvain‐La‐Neuve, Belgium) double scattering proton system at MGH (Massachusetts General Hospital, Boston, MA, USA). In that model, a relationship between the output and a single factor r=(R−M)/M, which is a function of the distal range, R, and modulation width, M, of the SOBP, was established. Kooy et al.^(2)^ improved their model by adding a correction factor that takes into account the shift of the effective source position as a function of proton distal range due to the change of fixed scattering materials. Engelsman et al.^(12)^ further refined the model by redefining the modulation width to be the distance between the proximal 98% dose level and the distal 90% dose level, rather than between the proximal and distal 90% dose levels, as the position of the proximal 98% point is well defined and has less uncertainty than the position of the proximal 90% point. In the current implementation, the MGH group can predict outputs to within 1.4% (one SD) of measurements^12^, ^13^, ^14^

As the University of Pennsylvania (UPenn) uses an IBA proton therapy system that is similar to the one at MGH, there was interest in commissioning the MGH model in our clinic. The main difference between the MGH and the UPenn systems as it pertains to output stems from the MGH group's freedom to adjust the beam current modulation (BCM) of their system. While this enables the MGH group both to fine‐tune the flatness of their SOBP distributions and to bring measured modulation widths into line with nominal modulation widths,^13^, ^14^ this is not something that is permissible contractually on the UPenn system, nor on IBA systems installed elsewhere. Due to this, we found that desirable output prediction accuracy could not be achieved by applying the MGH model directly to the UPenn proton system. To apply the methodology to our center (and, by extension, to others), it is necessary to introduce a linear‐quadratic transformation from the nominal modulation width to the measured modulation width. In this paper, we describe a method to overcome the problem that arises when implementing the MGH‐type semiempirical MU calculation procedure if these two widths differ appreciably. We first present how the model parameters are determined from limited measurements of systematic outputs, and then compare outputs predicted by this extended model with patient‐specific field measurements.

Cases, although IBA can generate flat SOBPs to make the dose at this point fall within 88% to 92% dose for typical modulation widths. For instance, Table 1 shows, for an example beam range of 17.5 cm, that measured and nominal modulation widths agree to within 2 mm for 5 and 10 cm nominal modulation widths across the three proton double scattering beamlines at our facility (named P1, P4, and P5), but that there are marked differences for 2 cm and full modulation widths. For the shortest modulation width, this translates into ~18% interbeamline variation in output (Table 1). By comparison, the consistency of measured range and output within the same day is better than 0.5 mm and 0.5%, respectively. Moreover, just as the Eclipse treatment planning system (Varian Medical Systems, Inc., Palo Alto, CA) can be configured to account for the difference between nominal and measured modulation widths,^(15)^ we seek to relate these two widths for the purpose of output prediction. We propose to do so through a linear‐quadratic model:

(1) $$r_{\mathrm{model}}=b_{2}r_{\mathrm{nominal}}^{2}+b_{1}r_{\mathrm{nominal}}+b_{0}$$

**Table 1:** Various measured modulation widths and outputs for beams of range 17.5 cm in three different treatment beamlines (P1, P4, and P5)

*Nomjnal M (cm)*	*2*	*5*	*10*	*17.5*
Measured *M* ‐ P1 (cm)	1.88	4.9	10.08	16.27
Measured *M* ‐ P4 (cm)	3.20	5.08	10.03	17.12
Measured *M* ‐ P5 (cm)	3.12	4.86	10.16	16.97
Output ‐ P1 (cGy/MU)	1.88	1.376	1.084	0.925
Output ‐ P4 (cGy/MU)	1.587	1.370	1.084	0.900
Output ‐ P5 (cGy/MU)	1.627	1.395	1.088	0.904

where $r_{\mathrm{nominal}}$ is related to a beam's nominal range, R, and nominal modulation width, M, via

(2) $$r_{\mathrm{nominal}}=\frac{R-0.91*M}{0.91*M}$$

The constant 0.91 in Eq. (2) is a theoretical value used for converting our definition of modulation width (proximal 90% to distal 90%) to the original definition of proximal 100% to distal 100% by Bortfeld and Schlegel,^(11)^ and was derived according to Eq. 8 in their paper. By propagating the MGH model, the output at the center of the SOBP is then given as

(3) $$\psi =CF\times (1+a_{1}r_{\mathrm{model}}^{a_{2}})\times [1+s\times (R-R_{m})]$$

where *CF* is a constant to correct for the output change per option, 5 is a fit parameter to account for the variation of effective SAD within a beam option, and $R_{m}$ is the minimal range of the option. Equations (2) and (3) follow from the work performed at MGH;^1^, ^2^ Eq. (1) is newly formulated here. Coefficients $b_{0}$, $b_{1}$, and $b_{2}$ are to be determined for each beam option using reference beams of the midrange suboption. Maximal $R^{2}$ or minimal residual sum of squares of the difference between the fitted and measurement data are used to determine the optimal parameters. After $b_{0}$, $b_{1}$, and $b_{2}$ are determined, $a_{1}$ and $a_{2}$ parameters are derived using the midrange suboption reference beams. However, when the midrange suboption's output dependence on modulation width does not represent that of the other two suboptions within an option, model parameters must be derived per suboption in order to fit the measured output data to within 2%. After $b_{0}$, $b_{1}$, $b_{2}$, $a_{1}$, and $a_{2}$ are determined, parameters *s* and *CF* are subsequently determined for the source position change with beam range and overall output constant using all the reference beams in all the three suboptions of each beam option.

Table 2 lists 60 reference beams that were used to derive the model coefficients in Eqs. (1) and (3). For each option, reference ranges were chosen at the two extremes and approximately midway between. For the minimal and maximal ranges, reference modulation widths (10 cm for B5‐B8, 5 cm for B2‐B4, and 3 cm for B1) were selected, while for the midrange suboption several modulation widths from 2 cm to full modulation width were utilized. Of these 60 beams, 47 were used initially to fit for the model parameters. We observed that dedicated fitting of the B2 and B6 suboptions was necessary to achieve 2% output accuracy. Therefore, 13 additional beams from the B2 and B6 low‐ and high‐range suboptions are included for the fitting. The performance of the output model was initially validated with 28 reference beams from low‐ and high‐range suboptions of B1, B3, B4, B5, B7, and B8 options (Table 3) and subsequently tested with 1784 patient‐specific fields.

**Table 2:** 60 reference beams used to derive the beamline‐specific and option‐specific MGH model parameters. SOBP RxMy has range × cm and modulation y cm. A span of SOBP is called beam option B#. A subspan within an option is called a “suboption” and is designated by the suffix _1, _2 or _3 (e.g., B1_1, etc.)

B1	R5M2	R5M3	R5M4	R5M5	R4.6M3	R5.86M3
B2_1	R5.87M2^a^	R5.87M3	R5.87M5^a^			
B2_2	R6.5M2	R6.5M3	R6.5M5	R6.5M6		
B2_3	R7.49M2^a^	R7.49M3	R7.49M5^a^	R7.49M6.5^a^		
B3	R8.5M2	R8.5M5	R8.5M8.5	R7.5M5	R9.54M5	
B4	R10.5M2	R10.5M5	R10.5M10.5	R9.55M5	R11.85M5	
B5	R13.5M2	R13.5M5	R13.5M10	R13.5M13.5	R11.86M10	R15.53M10
B6_1	R15.54M2^a^	R15.54M3^a^	R15.54M5^a^	R15.54M10	R15.54M14.5^a^	
B6_2	R17.5M2	R17.5M3	R17.5M5	R17.5M10	R17.5M17.5	
B6_3	R19.83M2^a^	R19.83M3^a^	R19.83M5^a^	R19.83M10	R19.83M18^a^	
B7	R22M2	R22M5	R22M10	R22M15	R19.84M10	R23.91M10
B8	R25M2	R25M5	R25M10	R25M15	R22.8M10	R28.26M10

a These 13 beams are used for better fitting of the B2 and B6 suboptions.

**Table 3:** Twenty‐eight reference beams of the B1, B3, B4, B5, B7, and B8 suboptions with extreme range and modulations used to validate the model parameters derived from Table 2

B1_1	R4.6M2	R4.6M4	B4_1	R9.55M2	R9.55M9	B7 1	R19.84M2	R19.84M5	R19.84M15
B1_3	R5.86M2	R5.86M5	B4_3	R11.85M2	R11.85M10	B7 3	R23.91M2	R23.91M5	R23.91M15
B3_1	R7.5M2	R7.5M6.5	B5 1	R11.86M2	R11.86M5	B8_1	R22.8M2	R22.8M5	R22.8M15
B3_3	R9.54M2	R9.54M9	B5 3	R15.53M2	R15.53M5	B8_3	R28.26M2	R28.26M5	R28.26M15

Output measurements were made in a water phantom with SAD geometry using a PPC05 ionization chamber (IBA Dosimetry, Schwarzenbruck, Germany) aligned to isocenter and the center of the SOBP. Previous reports have investigated the output dependence on field size and snout position^5^, ^7^, ^16^ and therefore the dependence of output on field size or snout position is not reported in this paper. Instead, a $10\times 10\;{\text{cm}}^{2}$ field and an air gap of 15 cm were used in this work to represent the average scatter condition, which minimizes the discrepancies introduced by the difference of field size and snout position in patient fields from the reference conditions. We restrict our application of the model to patient fields above $5\times 5\;{\text{cm}}^{2}$, as the measured outputs of smaller field size would often fall below 2% of the modeled outputs and need patient‐specific measurement. As a nominal rate of 2 Gy per minute is always used for our double scattering delivery, dose rate dependence was not investigated.

## III. RESULTS

**Figure 1:**
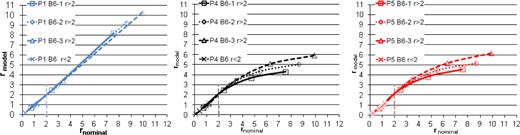
Linear‐quadratic model to convert nominal to measured modulation widths. P1 (blue lines, left), P4 (black lines, middle), P5 (red lines, right) refer to three different beamlines at our facility. B6_1 (square marker), B6_2 (diamond marker) and B6_3 (triangle marker) are three suboptions of the B6 option. The variable $r_{\text{nominal}}$ is divided at a value of 2 to convert large and small modulation widths using different fit values for parameters $b_{0}$, $b_{1}$, and $b_{2}$. B6 (r<2) curves are shown with “x” markers, and are identical for all three suboptions of a given beamline.

Tables 4, 5, and 6 displays the model coefficients extracted from Eqs. (1) and (3) for each option or suboption of the three beamlines. It was found that the coefficients of Eq. (1) had to be derived separately among the suboptions of B2 and B6 in each case in order to achieve output accuracy within 2%, but that suboption‐specific parameterizations were not required for the other options. Depending on the sign of $b_{2}$, $r_{\text{model}}$ will depart upward or downward from the linear relationship with $r_{\text{nominal}}$ (Fig. 1) and this upward or downward departure could be different for large and small modulation widths (small and large r, respectively). If the 2% accuracy of the fit of Eq. (3) could not be achieved for all modulation widths within an option or a suboption, $r_{\text{nominal}}$ was further broken into large and small modulation width components (options B5‐B8 (Tables 4, 5, 6)).

**Table 4:** Output model parameters of beamline P1

*Option*	*CF*	$a^{1}$	$a^{2}$	$b^{2}$	$b^{1}$	$b^{0}$	*s*	$R_{m}\;(cm)$
B1	0.677	0.4933	0.69177	0	0.93257	0.07286	0.0276	4.6
B2‐1	0.670	0.58345	0.61897	0	0.92578	0.01932	0.0300	5.86
B2‐2	0.689	0.57558	0.56474	0	0.97321	−0.00129	0.0000	5.86
B2‐3	0.711	0.57693	0.56369	0	0.96567	−0.03908	−0.0200	5.86
B3	0.735	0.50406	0.58053	0.0039	1.0107	0.0033	0.0126	7.49
B4	0.710	0.60838	0.49789	0.04189	0.86883	0.05977	0.0178	9.54
B5 r>2	0.685	0.51036	0.53197	−0.07516	1.25614	−0.14522	0.0173	11.86
B5 r<2	0.685	0.51036	0.53197	0.16743	0.65577	0.09761	0.0173	11.86
B6‐1 r<2	0.796	0.35963	0.58597	0.01187	0.99903	−0.00318	0.0000	15.53
B6‐1 r<2	0.796	0.35963	0.58597	0.06377	0.89519	0.02232	0.0000	15.53
B6‐2 r>2	0.819	0.35324	0.59653	0.00592	1.02821	−0.04525	0.0000	15.53
B6‐2 r<2	0.819	0.35324	0.59653	0.06377	0.89519	0.02232	0.0000	15.53
B6‐3 r>2	0.858	0.30171	0.62052	0.00215	1.01444	−0.02873	0.0000	15.53
B6‐3 r<2	0.858	0.30171	0.62052	0.06377	0.89519	0.02232	0.0000	15.53
B7 r>4	0.813	0.39906	0.52019	0.00356	0.95593	0.07888	0.0038	19.83
B7 r<4	0.813	0.39906	0.52019	−0.0024	0.99999	−0.00468	0.0038	19.83
B8 r>5	1.045	0.30657	0.58088	−0.02376	1.19746	−0.31829	0.0003	22.8
B8 r<5	1.045	0.30657	0.58088	0.05991	0.68095	0.31268	0.0003	22.8

**Table 5:** Output model parameters of beamline P4

*Option*	*CF*	$a_{1}$	$a_{2}$	$b_{2}$	$b_{1}$	$b_{0}$	s	$R_{m}$ (cm)
B1	0.674	0.51118	0.66454	0	0.9481	0.01087	0.0220	4.59
B2‐1	0.697	0.50521	0.66396	0	0.90502	0.04006	0.0000	5.86
B2‐2	0.697	0.52781	0.60559	0	0.97437	−0.00828	0.0000	5.86
B2‐3	0.717	0.58786	0.54393	0	0.98752	−0.04895	−0.0200	5.86
B3	0.720	0.53605	0.56672	−0.00086	0.99519	−0.01693	0.0063	7.49
B4	0.759	0.50127	0.57012	0.01839	0.92318	0.02303	0.0147	9.54
B5 r>2	0.719	0.42957	0.60077	−0.02428	1.072	−0.03795	0.0128	11.85
B5 r<2	0.719	0.42957	0.60077	0.01832	0.96442	0.00896	0.0128	11.85
B6‐1 r>2	0.819	0.32206	0.6867	−0.0848	1.21597	−0.07094	0.0000	15.53
B6‐1 r<2	0.819	0.32206	0.6867	0.0067	0.95273	0.01858	0.0000	15.53
B6‐2 r>2	0.820	0.3539	0.60336	−0.07662	1.25355	−0.11591	0.0000	15.53
B6‐2 r<2	0.820	0.3539	0.60336	0.0067	0.95273	0.01858	0.0000	15.53
B6‐3 r>2	0.821	0.36493	0.58221	−0.06746	1.28753	−0.23697	0.0000	15.53
B6‐3 r<2	0.821	0.36493	0.58221	0.0067	0.95273	0.01858	0.0000	15.53
B7 r>4	0.849	0.30623	0.60989	−0.02784	1.1038	−0.05817	0.0100	19.83
B7 r<4	0.849	0.30623	0.60989	0.01698	0.83954	0.1949	0.0100	19.83
B8 r>5	1.036	0.30291	0.58154	−0.00627	0.97666	0.08779	0.0006	22.79
B8 r<5	1.036	0.30291	0.58154	−0.00221	0.95563	0.09909	0.0006	22.79

**Table 6:** Output model parameters of beamline P5

*Option*	*CF*	$a_{1}$	$a_{2}$	$b_{2}$	$b_{1}$	$b_{0}$	*s*	$R_{m}(cm)$
B1	0.680	0.51363	0.68618	0	0.95675	0.07746	0.0195	4.59
B2‐1	0.580	0.82451	0.47693	0	0.90435	0.0907	0.0000	5.86
B2‐2	0.690	0.5475	0.6355	0	0.95668	0.06037	0.0000	5.86
B2‐3	0.580	0.89078	0.44324	0	0.94757	0.0406	0.0000	5.86
B3	0.709	0.55578	0.54997	−0.00203	1.01644	−0.00972	0.0093	7.49
B4	0.735	0.53385	0.52836	0.05376	0.88384	0.05744	0.0125	9.54
B5 r>2	0.724	0.41085	0.61169	−0.01777	1.10467	−0.04213	0.0136	11.85
B5 r<2	0.724	0.41085	0.61169	0.05606	0.91636	0.04146	0.0136	11.85
B6‐1 r>2	0.820	0.33162	0.65112	−0.0852	1.25779	−0.10673	0.0000	15.53
B6‐1 r<2	0.820	0.33162	0.65112	0.0783	0.85616	0.03933	0.0000	15.53
B6‐2 r>2	0.830	0.3374	0.64023	−0.0779	1.29031	−0.17739	0.0000	15.53
B6‐2 r<2	0.830	0.3374	0.64023	0.0783	0.85616	0.03933	0.0000	15.53
B6‐3 r>2	0.850	0.32875	0.61282	−0.06484	1.28128	−0.20303	0.0000	15.53
B6‐3 r<2	0.850	0.32875	0.61282	0.0783	0.85616	0.03933	0.0000	15.53
B7 (r>4)	0.893	0.25340	0.64342	−0.0177	1.07926	−0.0645	0.0051	19.83
B7 (r<4)	0.893	0.25340	0.64342	0.04319	0.82214	0.06896	0.0051	19.83
B8 (r>5)	1.030	0.29083	0.58558	−0.00429	0.96277	0.2781	0.0002	22.79
B8 (r<5)	1.030	0.29083	0.58558	−0.04321	1.21205	−0.0557	0.0002	22.79

As an example, Fig. 1 shows the linear‐quadratic relationship between $r_{\text{nominal}}$ and $r_{\text{model}}$ for each suboption of B6 in beamlines P1, P4, or P5. $r_{\text{model}}$ is similar to $r_{\text{nominal}}$ when $r_{\text{nominal}}$ is smaller than 2 (i.e., large modulation width cases (M/R>~37%)). However, these terms diverge from one another for the different beamlines and different suboptions when $r_{\text{nominal}}$ is larger than 2 (i.e., in the case of small modulation widths (M/R<~37%)). Further, it can be observed that measured and nominal modulation widths were well matched by the vendor for all three suboptions in beamline P1, but that there was imperfect optimization for beamlines P4 and P5, and also variation by suboption. Without the linear‐quadratic transformation of nominal to measured modulation width (Eq. (1)), R^2^ between the linear‐fit $r_{\text{model}}$ and the nominal $r_{\text{nominal}}$ was above 99% for P1, but between 89% and 91% for P4 and P5. Using the linear‐quadratic fit, all the $R^{2}$ are above 99%.

**Figure 2:**
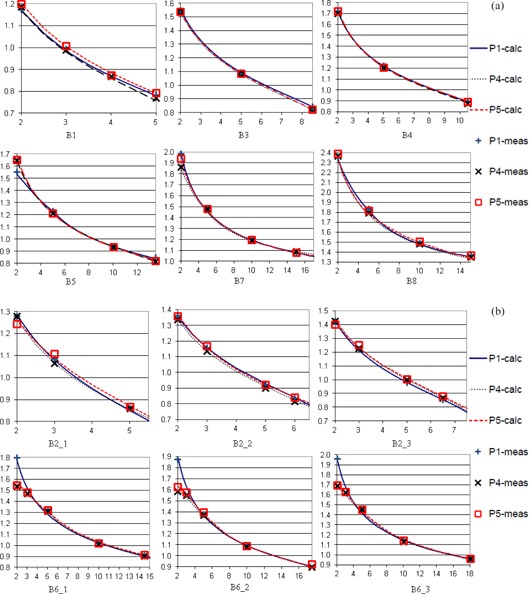
Comparison of models among three beamlines for each of the options B1, B3, B4, B5, B7 and B8 (a) and for the suboptions B2 and B6 (b). Blue solid lines stand for P1, black dotted lines stand for P4 and red dash lines stand for P5.

Figure 2(a) shows the fit of output to nominal modulation width for the reference beams of each of options B1, B3, B4, B5, B7, and B8 in all beamlines; Fig. 2(b) shows the fit for the three suboptions of both B2 and B6. From these, the necessity for per‐beamline modeling of the output can be seen. For instance, for option B5, P1 has significantly smaller outputs than P4 and P5 for small modulation widths, while for all suboptions of B6 the converse is true. This is because P1 measured modulation widths are longer than nominal values for option B5 beams when the modulation width is smaller than ~3 cm (hence higher MUs are required for the same mid‐SOBP dose), and because P4 and P5 measured modulation widths are longer than nominal values for option B6 beams when the modulation width is smaller than ~ 4 cm. Other suboptions also show interbeamline variations in output over some or all of the span of modulation widths. The maximal 7% (B5) and 18% (B6) interbeamline output variation at small modulation can be modeled individually to within 2% of measurement for each beam option (B5) or each suboption (B6) by using linear‐quadratic correction. The measured modulations are close to the nominal values for P4 and P5 of B5 option and P1 of B6 option. Without a linear‐quadratic correction of the nominal modulation width to the measured modulation width, the difference of modeled and measured B5 outputs in P1 and B6 outputs in P4 and P5 at small modulation can be modeled below the 7% and 18% interbeamline output variation, but at the expense of disagreement at medium and large modulation.

**Figure 3:**
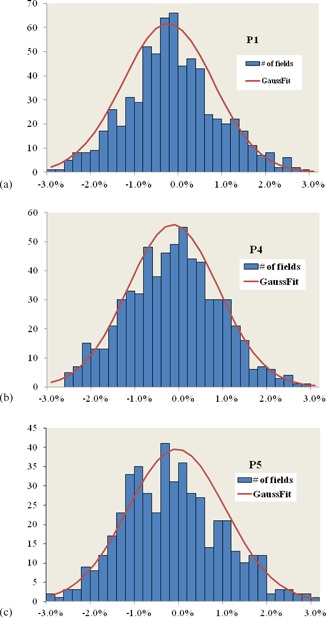
Percent difference between modeled and measured outputs for the three beamlines: (a) 662 fields with −0.12%±1.0%; (b) 647 fields with −0.13%±1.0%; (c) 475 fields with −0.14%±1.1%.

Figure 3 shows the difference between the model output prediction and measurement for 1784 patient‐specific fields for beamlines P1, P4, and P5. The modeled output is within 2% of the measurements for more than 95% of these fields, and for only two of these fields does it exceed 3% (one with 3.05% in P5 and the other with ‐3.07% in P1). The distribution of the fields amongst options is shown in Table 7. Because P1 treats primarily brain tumors and pediatric patients, B4 and B5 are dominant options, whereas in P5 where most treatments are for prostate cancer, B8 is dominant.

**Table 7:** Number of fields measured for each option of each beamline

	*B1*	*B2*	*B3*	*B4*	*B5*	*B6*	*B7*	*B8*	*Total*
P1	13	46	93	221	134	72	19	64	662
P4	13	30	62	115	147	78	42	160	647
P5	9	15	16	34	72	91	52	186	475
Basic	6	11	5	5	6	15	6	6	60

## IV. DISCUSSION

We described a procedure to implement the MGH model for calculating the output of proton double‐scattered beams as a function of range and measured modulation width. Since the nominal and measured modulation widths on our systems are different due to limitations in the vendor's ability to establish this correspondence over the full span of modulation widths for all beam ranges, we introduced quadratic parameters $b_{0}$, $b_{1}$, and $b_{2}$ to take into account this difference. All the model parameters can be derived from the 60 reference beams listed in Table 2. Subsequent measurements of 1784 patient‐specific field outputs demonstrate agreement to within 3% of the model prediction with <1.1% standard deviation. Such a good agreement for a large cohort of patient fields is at least comparable to the best results reported by the MGH group (1.4% standard deviation), and highly suggestive that the MGH output model can be implemented at institutions that may not have full control of how the SOBP is achieved. The additional parameters $b_{0}$, $b_{1}$ and $b_{2}$ model imperfections in the SOBP, which were unexpected in the MGH model, as parameters $a_{1}$ $a_{2}$, s, and CF are fundamentally related to the original formulation with empirical corrections.^(11)^

## V. CONCLUSIONS

A linear‐quadratic transformation of the nominal to the measured modulation width is essential to the clinical implementation of the MGH MU calculation model in order to account for imperfectly matched SOBP widths and achieve 3% output prediction accuracy. A method to derive the linear‐quadratic coefficients $b_{0}$, $b_{1}$, and $b_{2}$ is established.

## ACKNOWLEDGMENTS

The author Liyong Lin was a trainee at the University of Florida Proton Institute and learned the MGH‐type MU model from Roelf Slopsema.

## Supporting information

Supplementary Material
