# Supplementary material for: Implementation of an improved dose‐per‐MU model for double‐scattered proton beams to address interbeamline modulation width variability
Source: J Appl Clin Med Phys. 2014 May 8;15(3):297–306. doi: 10.1120/jacmp.v15i3.4748 (PMC5711055; doi:10.1120/jacmp.v15i3.4748)
Supplement: Supplementary file 1 — Supplementary Material [file ACM2-15-297-s001.doc]

Implementation of an improved dose-per-MU model for double scattered proton beams to address inter-beamline modulation width variability

**Abstract:** Because treatment planning systems (TPSs) generally do not provide monitor units (MUs) for double-scattered proton plans, models to predict MUs as a function of the range and the nominal modulation width requested of the beam delivery system, such as the one developed by the MGH group, have been proposed. For a given nominal modulation width, however, the measured modulation width depends on the accuracy of the vendor’s calibration process and may differ from this nominal value, and also from one beamline to the next. Although such a difference can be replicated in our TPS, the output dependence on range and modulation width for each beam option or sub-option has to be modeled separately for each beamline in order to achieve 3% accuracy. As a consequence, the MGH group’s output model may not be directly transferable. This work therefore serves to extend the model to more general clinic situations.

In this paper, a parameterized linear-quadratic transformation is introduced to convert the nominal modulation width to the measured modulation width for each beam option or sub-option on a per-beamline basis. Fit parameters are derived for each beamline from measurements of 60 reference beams spanning the minimum and maximum ranges, and modulation widths from 2 cm to full range per option or sub-option. Using the modeled modulation width, we extract the MGH parameters for the output dependence on range and modulation width. Our method has been tested with 1784 patient-specific fields delivered across three different beamlines at our facility. For these fields, all measured outputs fall within 3%, and 64.4% fall within 1%, of our model.

Using a parameterized linear-quadratic modulation width, MU calculation models can be established on a per-beamline basis for each double-scattering beam option or sub-option.

| PACS numbers*: 87.53.Qc* |  |
| --- | --- |

Key Words: proton therapy, double scattering, output model

1. **Introduction**

It is well known that proton therapy has the advantage of sparing normal tissue because of the finite proton range. The clinical utilization of proton therapy requires safe and efficient planning and delivery technologies. However, the calculation of output (dose/MU) is not supported by commercial proton therapy treatment planning systems (TPSs) due to the complexities of the beam delivery systems. Historically, output was therefore determined by measurement for each field prior to treatment. Although adequate, this undertaking unfortunately requires a significant amount of beam time and manpower.

  Output calculation methods for different proton therapy systems, using either empirical models ^(1-7)^ or Monte Carlo simulations ^(8-10)^, have been described in several publications. These methods have been used for MU determination and/or as independent checks of measured output. Although less sophisticated, the advantage of empirical models over Monte Carlo simulations is their explicit form.

  An analytical expression for the depth-dose of an SOBP at infinite SAD was derived by Bortfeld and Schlegel in 1996 ^(11)^. Kooy et al 2003 ^(1)^ extended Bortfeld and Schlegel’s analysis to a specific model for the IBA (Ion Beam Applications, SA, Louvain-La-Neuve, Belgium) double scattering proton system at MGH (Massachusetts General Hospital, Boston, MA, USA). In that model, a relationship between the output and a single factor *r* = (*R* − *M*)/*M*, which is a function of the distal range, *R*, and modulation width, *M*, of the spread-out Bragg peak (SOBP) was established. Kooy et al. 2005 ^(2)^ improved their model by adding a correction factor that takes into account the shift of the effective source position as a function of proton distal range due to the change of fixed scattering materials. Engelsman et al 2009 ^(12)^ further refined the model by redefining the modulation width to be the distance between the proximal 98% dose level and the distal 90% dose level, rather than between the proximal and distal 90% dose levels, as the position of the proximal 98% point is well-defined and has less uncertainty than the position of the proximal 90% point. In the current implementation, the MGH group can predict outputs to within 1.4% (one SD) of measurements. ^(12-14)^

  As the xx uses an IBA proton therapy system that is similar to the one at MGH, there was interest in commissioning the MGH model in our clinic. The main difference between the MGH and UPenn systems as it pertains to output stems from the MGH group’s freedom to adjust the beam-current-modulation (BCM) of their system. While this enables the MGH group both to fine-tune the flatness of their SOBP distributions and to bring measured modulation widths into line with nominal modulation widths (Lu et al 2006, 2007^13-14)^), this is not something that is permissible contractually on the xx system, nor on IBA systems installed elsewhere. Due to this, we found that desirable output prediction accuracy could not be achieved by applying the MGH model directly to the xx proton system. To apply the methodology to our center (and, by extension, to others), it is necessary to introduce a linear-quadratic transformation from the nominal modulation width to the measured modulation width. In this paper, we describe a method to overcome the problem that arises when implementing the MGH-type semi-empirical MU calculation procedure if these two widths differ appreciably. We first present how the model parameters are determined from limited measurements of systematic outputs, and then compare outputs predicted by this extended model with patient-specific field measurements.

1. **Methods and Materials**

The IBA double scattering technique utilizes eight treatment “options”, designated B1–B8, each of which is defined by a unique combination of second scatterer, range modulator wheel track and BCM, and is applicable to a limited span of beam ranges. In order to be able to use a range modulator wheel track over a wide proton energy range, IBA applies different BCM for three sub-spans of range within an option. Such a sub-span within an option is called a “sub-option” and is designated by the suffix _1, _2 or _3 (e.g., B1_1, etc.).

The MGH MU model is derived fundamentally from SOBPs of ideal modulation width, where nominal and measured values are identical, and is not therefore directly applicable to practical situations where these values differ. In each IBA beamline, a polynomial fit is performed by the vendor prior to customer acceptance that relates the window of time during which the beam is on as the modulator wheel rotates to the resulting modulation width. Where these fits perform less well, differences in the nominal (*i.e.*, fitted) and measured widths can exceed 1 cm in length or 3% in dose at the nominal proximal 90% dose point in some extreme cases, although IBA can generate flat SOBPs to make the dose at this point fall within 88% to 92% dose for typical modulation widths. For instance, Table 1 shows, for an example beam range of 17.5 cm, that measured and nominal modulation widths agree to within 2 mm for 5 and 10 cm nominal modulation widths across the three proton double scattering beamlines at our facility (named P1, P4 and P5), but that there are marked differences for 2 cm and full modulation widths. For the shortest modulation width, this translates into ~18% inter-beamline variation in output (Table 1). By comparison, the consistency of measured range and output within the same day is better than 0.5 mm and 0.5%, respectively. Moreover, just as the Eclipse treatment planning system (Varian Medical Systems, Inc., Palo Alto, CA) can be configured to account for the difference between nominal and measured modulation widths (Ainsley 2013^(15)^), we seek to relate these two widths for the purpose of output prediction. We propose to do so through a linear-quadratic model:

$r_{model}=b_{2}r_{nominal}^{2}+b_{1}r_{nominal}+b_{0}$, (1)

where *r_nominal_* is related to a beam's nominal range, *R*, and nominal modulation width, *M*, via

$r_{nominal}=\frac{R-0.91*M}{0.91*M}$. (2)

The constant 0.91 in Equation 2 is a theoretical value used for converting our definition of modulation width (proximal 90% to distal 90%) to the original definition of proximal 100% to distal 100% by Bortfeld and Schlegel ^(11)^, and was derived according to Equation 8 in their paper. By propagating the MGH model, the output at the center of the SOBP is then given as

$\Psi=CF\times(1+a_{1}r_{model}^{a_{2}})\times[1+s\times(R-R_{m})]$, (3)

where *CF* is a constant to correct for the output change per option, *s* is a fit parameter to account for the variation of effective SAD within a beam option and *R_m_* is the minimal range of the option. Equations 2 and 3 follow from the work performed at MGH ^(1-2)^; Equation 1 is newly formulated here. Coefficients *CF*, *a*_1_, *a*_2_, *b*_0_, *b*_1_, *b*_2_ and *s* are to be determined.

Table 2 lists 60 reference beams that were used to derive the model coefficients in Equations 1 and 3. For each option, reference ranges were chosen at the two extremes and approximately midway between. For the minimal and maximal ranges, reference modulation widths (10 cm for B5-B8, 5 cm for B2-B4 and 3 cm for B1) were selected, while for the mid-option range several modulation widths from 2 cm to full modulation width were utilized. Of these 60 beams, 47 (not underlined in Table 2a) were used initially to fit for the model parameters. However, when the mid-option range's output dependence on modulation width does not represent that of the other two sub-options within an option, model parameters must be derived per sub-option in order to fit the measured output data to within 2%. We observed that dedicated fitting of the B2 and B6 sub-options was necessary to achieve this level of accuracy. Therefore, 13 additional beams (underlined in Table 2a) from the B2 and B6 low- and high-range sub-options are included for the fitting. The performance of the output model was subsequently tested with 1784 patient-specific fields.

Output measurements were made in a water tank with SAD geometry using a PPC05 (IBA Dosimetry, Schwarzenbruck, Germany) ionization chamber aligned to isocenter and the center of the SOBP. All beams were delivered with a dose rate of 2 Gy min^−1^. In all cases, an MLC field size of 10 × 10 cm^2^ was employed to represent average scatter conditions. For beams of given range and modulation, outputs for all clinical field sizes from 5 × 5 to 15 × 15 cm^2^ were found to vary by < ±1% from their values for a 10 × 10 cm^2^ field. We restrict out application of the model to field sizes within this range, and do not discuss the output dependence on field size further in this paper.

1. **Results**

Table 3 displays the model coefficients extracted from Equations 1 and 3 for each option or sub-option of the three beamlines. It was found that the coefficients of Equation 1 had to be derived separately among the sub-options of B2 and B6 in each case in order to achieve output accuracy within 2%, but that sub-option-specific parameterizations were not required for the other options. Depending on the sign of *b*_2_, *r_model_* will depart upward or downward from the linear relationship with *r_nominal_* (Figure 1) and this upward or downward departure could be different for large and small modulation widths (small and large *r*, respectively). If the 2% accuracy of the fit of Equation 3 could not be achieved for all modulation widths within a sub-option, *r_nominal_* was further broken into large and small modulation width components (options B5–B8 (Table 3)).

As an example, Figure 1 shows the linear-quadratic relationship between *r_nominal_* and *r_model_* for each sub-option of B6 in beamlines P1, P4 or P5. *r_model_* is similar to *r_nominal_* when *r_nominal_* is smaller than 2, *i.e.*, large modulation width cases (*M* / *R* > ~37%). However, these terms diverge from one another for the different beamlines and different sub-options when *r_nominal_* is larger than 2, *i.e.*, in the case of small modulation widths (*M* / *R* < ~37%). Further, it can be seen that measured and nominal modulation widths were well matched by the vendor for all three sub-options in beamline P1, but that there was imperfect optimization for beamlines P4 and P5, and also variation by sub-option. Without the linear-quadratic transformation of nominal to measured modulation width (Equation 1), deviations between the measured output and the fit prediction from Equation 3 (with *r_model_* replaced by *r_nominal_*) were found to exceed 5%.

Figure 2a shows the fit of output to nominal modulation width for the reference beams of each of options B1, B3, B4, B5, B7 and B8 in all beamlines; Figure 2b shows the fit for the three sub-options of both B2 and B6. From these, the necessity for per-beamline modeling of the output can be seen. For instance, for option B5, P1 has significantly smaller outputs than P4 and P5 for small modulation widths, while for all sub-options of B6 the converse is true. This is because P1 measured modulation widths are longer than nominal values for option B5 beams when the modulation width is smaller than ~3 cm (hence higher MUs are required for the same mid-SOBP dose), and because P4 and P5 measured modulation widths are longer than nominal values for option B6 beams when the modulation width is smaller than ~4 cm. Other sub-options also show inter-beamline variations in output over some or all of the span of modulation widths.

Figure 3 shows the difference between the model output prediction and measurement for 1784 patient-specific fields for beamlines P1, P4 and P5. The modeled output is within 2% of the measurements for more than 95% of these fields, and for only two of these fields does it exceed 3% (one with 3.05% in P5 and the other with -3.07% in P1). Without a linear-quadratic correction of the nominal modulation width to the measured modulation width, differences can be well above 5%. The distribution of the fields amongst options is shown in Table 4. Because P1 treats primarily brain tumors and pediatric patients, B4 and B5 are dominant options, whereas in P5 where most treatments are for prostate cancer, B8 is dominant.

1. **Discussion**

We described a procedure to implement the MGH model for calculating the output of proton double scattered beams as a function of range and measured modulation width. Since the nominal and measured modulation widths on our systems are different due to limitations in the vendor’s ability to establish this correspondence over the full span of modulation widths for all beam ranges, we introduced quadratic parameters *b*_0_, *b*_1_ and *b*_2_ to take into account this difference. All the model parameters can be derived from the 60 reference beams listed in Table 2. Subsequent measurements of patient-specific field outputs demonstrate agreement to within 3% of the model prediction.

Previous reports have investigated the output dependence on field size and snout position (Daartz et al 2009^(16)^, Zhao et al 2010^(5)^, Zheng et al 2011^(7)^); in this work a 10 × 10 cm^2^ field size and an air gap of 15 cm were used. This choice of reference field size and snout position largely minimizes the discrepancies introduced as a result, and residual contributions to the differences in output between the model prediction and measurement do not exceed 2% for field sizes larger than 5 × 5 cm^2^ for the 60 reference beams and for the majority of patient fields. As a consequence, the dependence of output on field size or snout position has not been extensively characterized in this paper. Dose rate dependence was not investigated as a nominal rate of 2 Gy min^−1^ is always used for our double scattering delivery.

1. **Conclusion**

A linear-quadratic transformation of the nominal to the measured modulation width is essential to the clinical implementation of the MGH MU calculation model in order to account for imperfectly matched SOBP widths and achieve 3% output prediction accuracy. A method to derive the linear-quadratic coefficients *b*_0_, *b*_1_ and *b*_2_ is established.

Acknowledgement

The author xx was a trainee at the University of Florida Proton Institute and learned the MGH-type MU model from Roelf Slopsema.

Reference:

1. Kooy HM, Schaefer M, Rosenthal S, and Bortfeld T 2003 Monitor unit calculations for range-modulated spread-out Bragg peak fields *Phys Med Biol* **48** 2797–2808
2. Kooy HM, Rosenthal SJ, Engelsman M, Mazal A, Slopsema RL, Paganetti H, and Flanz JB 2005 The prediction of output factors for spread out proton Bragg peak fields in clinical practice *Phys Med Biol* **50** 5847–5856
3. Sahoo N, Zhu XR, Arjomandy B, Ciangaru G, Amos R, Wu R, and Gillin MT 2008 A procedure for calculation of monitor units for passively scattered proton radiotherapy beams *Med Phys* **35** 5088–97
4. Hsi W, Schreuder AN, Moyers MF, Allgower CE, Farr JB, and Mascia AE 2009 Range and modulation dependencies for proton beam dose per monitor unit calculations *Med Phys* **36** 634–41
5. Zhao Q, Wu H, Wolanski M, Pack D, Johnstone PA, Das I 2010 A sector-integration method for dose/MU calculation in a uniform scanning proton beam *Phys Med Biol* **55**(3), N87–N95
6. Kim D, Lim Y, Ahn S et al. 2011 Prediction of output factor, range, and spread-out Bragg peak for proton therapy, *Med Dosim*, **36**(2), 145-152
7. Zheng Y, Ramirez E, Mascia A et al. 2011 Commissioning of output factors for uniform scanning proton beams, *Med Phys* **38**(4), 2299-2306
8. Paganetti H 2006 Monte Carlo calculations for absolute dosimetry to determine machine outputs for proton therapy fields *Phys Med Biol* **51**, 2801–2812
9. Hérault J, Iborra N, Serrano B, Chauvel P 2007 Spread-out Bragg peak and monitor unit calculation with the Monte Carlo code MCNPX *Med Phys* **34**, 680–688
10. Titt U, Zheng Y, Vassiliev ON, Newhauser WD 2008 Monte Carlo investigation of collimator scatter of proton-therapy beams produced using the passive scattering method *Phys Med Biol* **53** 487–504
11. Bortfeld T, Schlegel W 1996 An analytical approximation of depth-dose distributions for therapeutic proton beam *Phys Med Biol* **41**, 1331-1339
12. Engelsman M, Lu HM, Herrup D et al. 2009 Commissioning a passive-scattering proton therapy nozzle for accurate SOBP delivery *Med Phys* **36**, 2172–2180
13. Lu HM, Kooy H 2006 Optimization of current modulation function for proton spread-out Bragg peak fields *Med Phys* **33**, 1281–1287
14. Lu HM, Brett R, Engelsman M, Slopsema R, Kooy H, Flanz J 2007 Sensitivities in the production of spread-out Bragg peak dose distributions by passive scattering with beam current modulation *Med Phys* **34**, 3844–3853
15. Ainsley C, Lin L, McDonough J 2013 Optimization of the modeling of longitudinal dose distributions for double-scattered proton beams in a commercially-available treatment planning system *Phys Med Biol* **58** (11) N145-N155
16. Daartz J, Engelsman M, Pagnetti H, Bussiere MR 2009 Field size dependence of the output factor in passively scattered proton therapy: Influence of range, modulation, air gap, and machine settings *Med Phys* **36**, 3205-3210
17. Fontenot JD, Newhauser W, Bloch C, White RA, Titt U, Starkchall G 2007 Determination of output factors for small proton therapy fields *Med Phys* **34**, 489-498


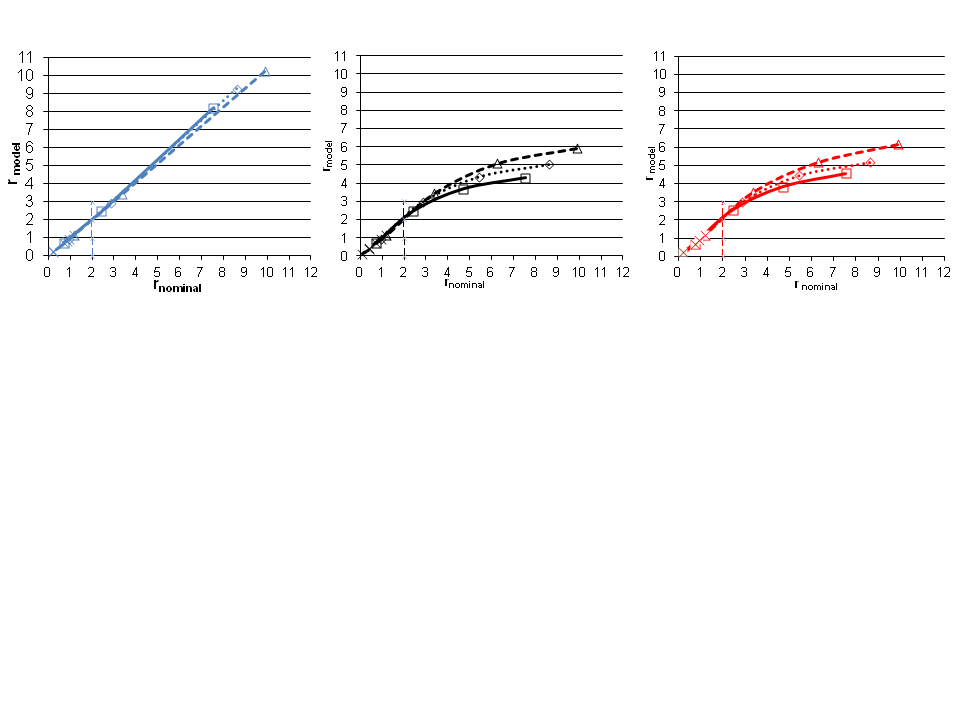


Figure 1: Linear-quadratic model to convert nominal to measured modulation widths. P1 (blue lines, left), P4 (black lines, middle), P5 (red lines, right) refer to three different beamlines at our facility. B6_1 (square marker), B6_2 (diamond marker) and B6_3 (triangle marker) are three sub-options of the B6 option. The variable *r_nominal_* is divided at a value of 2 to convert large and small modulation widths using different fit values for parameters *b*_0_, *b*_1_ and *b*_2_. B6 (r < 2) curves are shown with “x” markers, and are identical for all three sub-options of a given beamline.


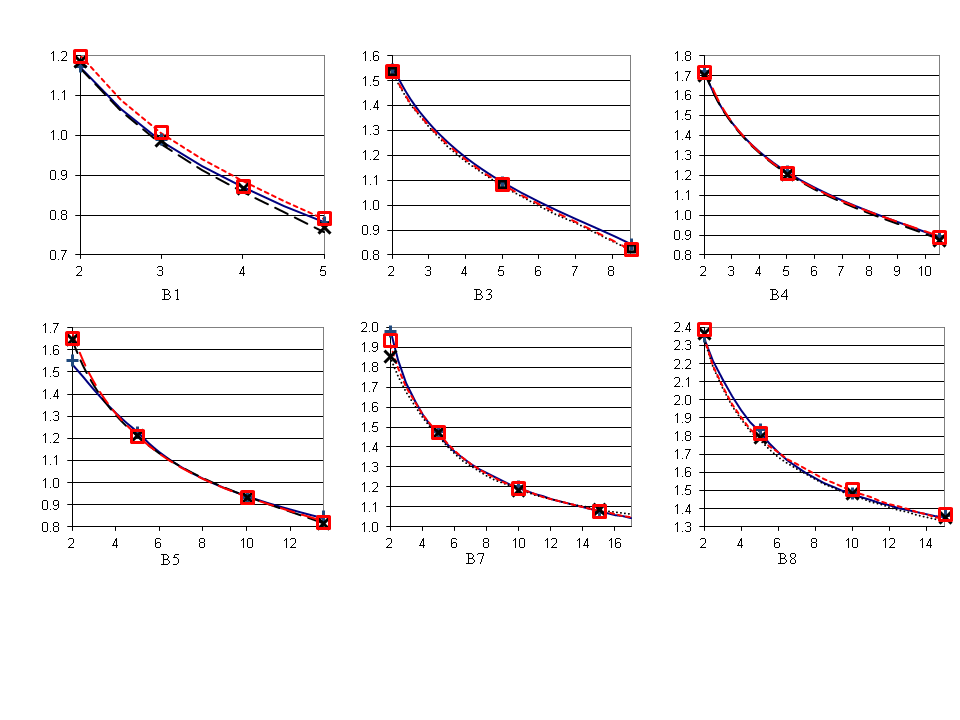


Figure 2a: Comparison of models among three beamlines for each of the options B1, B3, B4, B5, B7 and B8. Blue solid lines stand for P1, black dotted lines stand for P4 and red dash lines stand for P5.


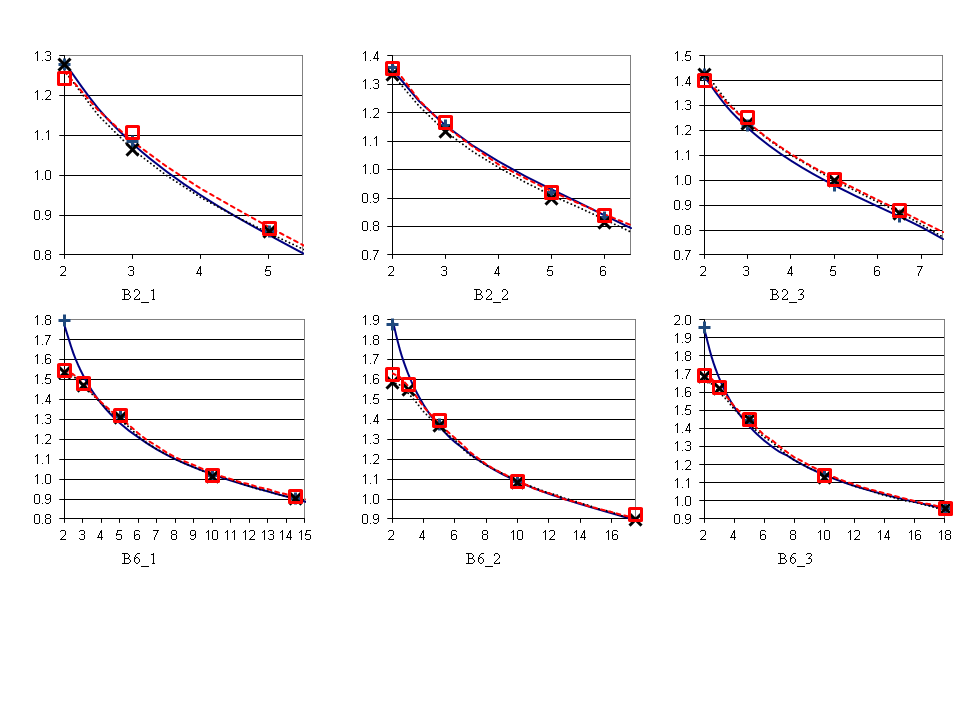


Figure 2b: Comparison of models among three beamlines for each of the sub-options of B2 and B6. Blue solid lines stand for P1, black dotted lines stand for P4 and red dash lines stand for P5.

| 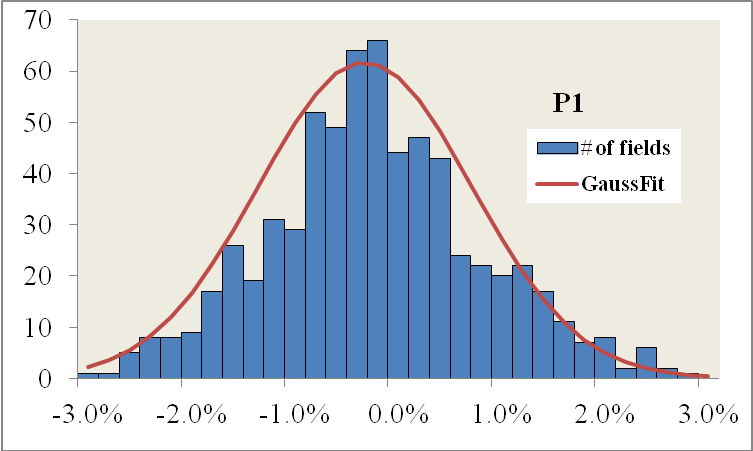 | 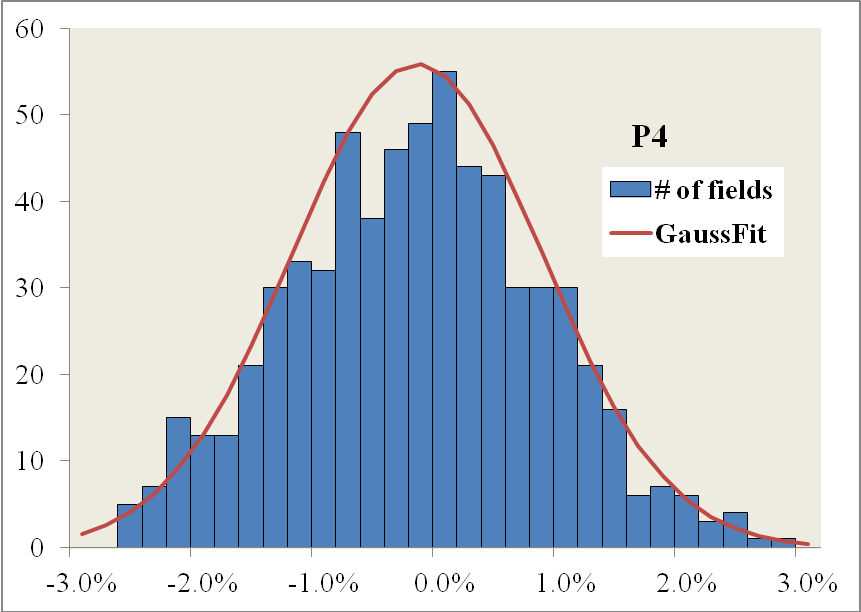 | 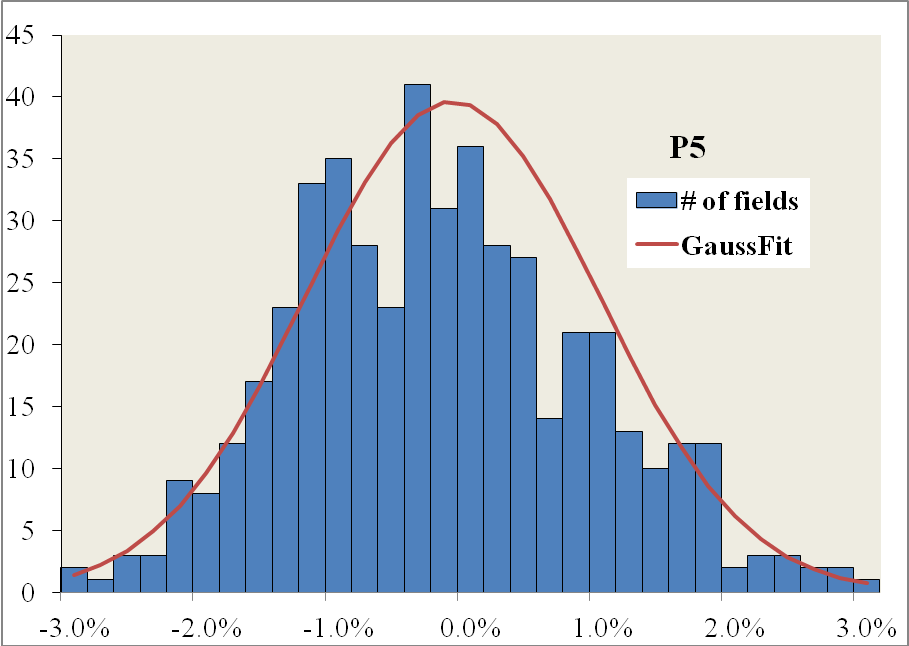 |
| --- | --- | --- |
| (a) 662 fields with -0.12%±1.0% | (b) 647 fields with -0.13%±1.0% | (c) 475 fields with -0.14%±1.1% |

Figure 3: Per-cent difference between modeled and measured outputs for the three beamlines.

Table 1: Various measured modulation widths and outputs for beams of range 17.5 cm in three different treatment beamlines (P1, P4 and P5).

| Nominal *M* (cm) | 2 | 5 | 10 | 17.5 |
| --- | --- | --- | --- | --- |
| Measured *M* - P1 (cm) | 1.88 | 4.9 | 10.08 | 16.27 |
| Measured *M* - P4 (cm) | 3.20 | 5.08 | 10.03 | 17.12 |
| Measured *M* - P5 (cm) | 3.12 | 4.86 | 10.16 | 16.97 |
| Output - P1 (cGy/MU) | 1.88 | 1.376 | 1.084 | 0.925 |
| Output - P4 (cGy/MU) | 1.587 | 1.370 | 1.084 | 0.900 |
| Output - P5 (cGy/MU) | 1.627 | 1.395 | 1.088 | 0.904 |

Table 2: 60 reference beams used to derive the beamline-specific and option-specific MGH model parameters. The 13 underlined beams are used for better fitting of the B2 and B6 sub-options.

| B1 | R5M2 | R5M3 | R5M4 | R5M5 | R4.6M3 | R5.86M3 |
| --- | --- | --- | --- | --- | --- | --- |
| B2_1 | R5.87M2 | R5.87M3 | R5.87M5 |  |  |  |
| B2_2 | R6.5M2 | R6.5M3 | R6.5M5 | R6.5M6 |  |  |
| B2_3 | R7.49M2 | R7.49M3 | R7.49M5 | R7.49M6.5 |  |  |
| B3 | R8.5M2 | R8.5M5 | R8.5M8.5 | R7.5M5 | R9.54M5 |  |
| B4 | R10.5M2 | R10.5M5 | R10.5M10.5 | R9.55M5 | R11.85M5 |  |
| B5 | R13.5M2 | R13.5M5 | R13.5M10 | R13.5M13.5 | R11.86M10 | R15.53M10 |
| B6_1 | R15.54M2 | R15.54M3 | R15.54M5 | R15.54M10 | R15.54M14.5 |  |
| B6_2 | R17.5M2 | R17.5M3 | R17.5M5 | R17.5M10 | R17.5M17.5 |  |
| B6_3 | R19.83M2 | R19.83M3 | R19.83M5 | R19.83M10 | R19.83M18 |  |
| B7 | R22M2 | R22M5 | R22M10 | R22M15 | R19.84M10 | R23.91M10 |
| B8 | R25M2 | R25M5 | R25M10 | R25M15 | R22.8M10 | R28.26M10 |

Table 3a: Output model parameters of beamline P1.

| Option | *CF* | *a_1_* | *a_2_* | *b_2_* | *b_1_* | *b_0_* | *s* | *R_m_* (cm) |
| --- | --- | --- | --- | --- | --- | --- | --- | --- |
| B1 | 0.677 | 0.4933 | 0.69177 | 0 | 0.93257 | 0.07286 | 0.0276 | 4.6 |
| B2-1 | 0.670 | 0.58345 | 0.61897 | 0 | 0.92578 | 0.01932 | 0.0300 | 5.86 |
| B2-2 | 0.689 | 0.57558 | 0.56474 | 0 | 0.97321 | -0.00129 | 0.0000 | 5.86 |
| B2-3 | 0.711 | 0.57693 | 0.56369 | 0 | 0.96567 | -0.03908 | -0.0200 | 5.86 |
| B3 | 0.735 | 0.50406 | 0.58053 | 0.0039 | 1.0107 | 0.0033 | 0.0126 | 7.49 |
| B4 | 0.710 | 0.60838 | 0.49789 | 0.04189 | 0.86883 | 0.05977 | 0.0178 | 9.54 |
| B5 r>2 | 0.685 | 0.51036 | 0.53197 | -0.07516 | 1.25614 | -0.14522 | 0.0173 | 11.86 |
| B5 r<2 | 0.685 | 0.51036 | 0.53197 | 0.16743 | 0.65577 | 0.09761 | 0.0173 | 11.86 |
| B6-1 r>2 | 0.796 | 0.35963 | 0.58597 | 0.01187 | 0.99903 | -0.00318 | 0.0000 | 15.53 |
| B6-1 r<2 | 0.796 | 0.35963 | 0.58597 | 0.06377 | 0.89519 | 0.02232 | 0.0000 | 15.53 |
| B6-2 r>2 | 0.819 | 0.35324 | 0.59653 | 0.00592 | 1.02821 | -0.04525 | 0.0000 | 15.53 |
| B6-2 r<2 | 0.819 | 0.35324 | 0.59653 | 0.06377 | 0.89519 | 0.02232 | 0.0000 | 15.53 |
| B6-3 r>2 | 0.858 | 0.30171 | 0.62052 | 0.00215 | 1.01444 | -0.02873 | 0.0000 | 15.53 |
| B6-3 r<2 | 0.858 | 0.30171 | 0.62052 | 0.06377 | 0.89519 | 0.02232 | 0.0000 | 15.53 |
| B7 r>4 | 0.813 | 0.39906 | 0.52019 | 0.00356 | 0.95593 | 0.07888 | 0.0038 | 19.83 |
| B7 r<4 | 0.813 | 0.39906 | 0.52019 | -0.0024 | 0.99999 | -0.00468 | 0.0038 | 19.83 |
| B8 r>5 | 1.045 | 0.30657 | 0.58088 | -0.02376 | 1.19746 | -0.31829 | 0.0003 | 22.8 |
| B8 r<5 | 1.045 | 0.30657 | 0.58088 | 0.05991 | 0.68095 | 0.31268 | 0.0003 | 22.8 |

Table 3b: Output model parameters of beamline P4.

| Option | *CF* | *a_1_* | *a_2_* | *b_2_* | *b_1_* | *b_0_* | *s* | *R_m_* (cm) |
| --- | --- | --- | --- | --- | --- | --- | --- | --- |
| B1 | 0.674 | 0.51118 | 0.66454 | 0 | 0.9481 | 0.01087 | 0.0220 | 4.59 |
| B2-1 | 0.697 | 0.50521 | 0.66396 | 0 | 0.90502 | 0.04006 | 0.0000 | 5.86 |
| B2-2 | 0.697 | 0.52781 | 0.60559 | 0 | 0.97437 | -0.00828 | 0.0000 | 5.86 |
| B2-3 | 0.717 | 0.58786 | 0.54393 | 0 | 0.98752 | -0.04895 | -0.0200 | 5.86 |
| B3 | 0.720 | 0.53605 | 0.56672 | -0.00086 | 0.99519 | -0.01693 | 0.0063 | 7.49 |
| B4 | 0.759 | 0.50127 | 0.57012 | 0.01839 | 0.92318 | 0.02303 | 0.0147 | 9.54 |
| B5 r>2 | 0.719 | 0.42957 | 0.60077 | -0.02428 | 1.072 | -0.03795 | 0.0128 | 11.85 |
| B5 r<2 | 0.719 | 0.42957 | 0.60077 | 0.01832 | 0.96442 | 0.00896 | 0.0128 | 11.85 |
| B6-1 r>2 | 0.819 | 0.32206 | 0.6867 | -0.0848 | 1.21597 | -0.07094 | 0.0000 | 15.53 |
| B6-1 r<2 | 0.819 | 0.32206 | 0.6867 | 0.0067 | 0.95273 | 0.01858 | 0.0000 | 15.53 |
| B6-2 r>2 | 0.820 | 0.3539 | 0.60336 | -0.07662 | 1.25355 | -0.11591 | 0.0000 | 15.53 |
| B6-2 r<2 | 0.820 | 0.3539 | 0.60336 | 0.0067 | 0.95273 | 0.01858 | 0.0000 | 15.53 |
| B6-3 r>2 | 0.821 | 0.36493 | 0.58221 | -0.06746 | 1.28753 | -0.23697 | 0.0000 | 15.53 |
| B6-3 r<2 | 0.821 | 0.36493 | 0.58221 | 0.0067 | 0.95273 | 0.01858 | 0.0000 | 15.53 |
| B7 r>4 | 0.849 | 0.30623 | 0.60989 | -0.02784 | 1.1038 | -0.05817 | 0.0100 | 19.83 |
| B7 r<4 | 0.849 | 0.30623 | 0.60989 | 0.01698 | 0.83954 | 0.1949 | 0.0100 | 19.83 |
| B8 r>5 | 1.036 | 0.30291 | 0.58154 | -0.00627 | 0.97666 | 0.08779 | 0.0006 | 22.79 |
| B8 r<5 | 1.036 | 0.30291 | 0.58154 | -0.00221 | 0.95563 | 0.09909 | 0.0006 | 22.79 |

Table 3c: Output model parameters of beamline P5.

| Option | *CF* | *a_1_* | *a_2_* | *b_2_* | *b_1_* | *b_0_* | *s* | *R_m_* (cm) |
| --- | --- | --- | --- | --- | --- | --- | --- | --- |
| B1 | 0.680 | 0.51363 | 0.68618 | 0 | 0.95675 | 0.07746 | 0.0195 | 4.59 |
| B2-1 | 0.580 | 0.82451 | 0.47693 | 0 | 0.90435 | 0.0907 | 0.0000 | 5.86 |
| B2-2 | 0.690 | 0.5475 | 0.6355 | 0 | 0.95668 | 0.06037 | 0.0000 | 5.86 |
| B2-3 | 0.580 | 0.89078 | 0.44324 | 0 | 0.94757 | 0.0406 | 0.0000 | 5.86 |
| B3 | 0.709 | 0.55578 | 0.54997 | -0.00203 | 1.01644 | -0.00972 | 0.0093 | 7.49 |
| B4 | 0.735 | 0.53385 | 0.52836 | 0.05376 | 0.88384 | 0.05744 | 0.0125 | 9.54 |
| B5 r>2 | 0.724 | 0.41085 | 0.61169 | -0.01777 | 1.10467 | -0.04213 | 0.0136 | 11.85 |
| B5 r<2 | 0.724 | 0.41085 | 0.61169 | 0.05606 | 0.91636 | 0.04146 | 0.0136 | 11.85 |
| B6-1 r>2 | 0.820 | 0.33162 | 0.65112 | -0.0852 | 1.25779 | -0.10673 | 0.0000 | 15.53 |
| B6-1 r<2 | 0.820 | 0.33162 | 0.65112 | 0.0783 | 0.85616 | 0.03933 | 0.0000 | 15.53 |
| B6-2 r>2 | 0.830 | 0.3374 | 0.64023 | -0.0779 | 1.29031 | -0.17739 | 0.0000 | 15.53 |
| B6-2 r<2 | 0.830 | 0.3374 | 0.64023 | 0.0783 | 0.85616 | 0.03933 | 0.0000 | 15.53 |
| B6-3 r>2 | 0.850 | 0.32875 | 0.61282 | -0.06484 | 1.28128 | -0.20303 | 0.0000 | 15.53 |
| B6-3 r<2 | 0.850 | 0.32875 | 0.61282 | 0.0783 | 0.85616 | 0.03933 | 0.0000 | 15.53 |
| B7 (r>4) | 0.893 | 0.25340 | 0.64342 | -0.0177 | 1.07926 | -0.0645 | 0.0051 | 19.83 |
| B7 (r<4) | 0.893 | 0.25340 | 0.64342 | 0.04319 | 0.82214 | 0.06896 | 0.0051 | 19.83 |
| B8 (r>5) | 1.030 | 0.29083 | 0.58558 | -0.00429 | 0.96277 | 0.2781 | 0.0002 | 22.79 |
| B8 (r<5) | 1.030 | 0.29083 | 0.58558 | -0.04321 | 1.21205 | -0.0557 | 0.0002 | 22.79 |

Table 4: Number of fields measured for each option of each beamline.

| # of fields | B1 | B2 | B3 | B4 | B5 | B6 | B7 | B8 | total |
| --- | --- | --- | --- | --- | --- | --- | --- | --- | --- |
| P1 | 13 | 46 | 93 | 221 | 134 | 72 | 19 | 64 | 662 |
| P4 | 13 | 30 | 62 | 115 | 147 | 78 | 42 | 160 | 647 |
| P5 | 9 | 15 | 16 | 34 | 72 | 91 | 52 | 186 | 475 |
| basic | 6 | 11 | 5 | 5 | 6 | 15 | 6 | 6 | 60 |
